# Computed tomography analysis of fascial space involvement demonstrates correlations with laboratory tests, length of hospital stays and admission to the intensive care unit in odontogenic infections

**DOI:** 10.1016/j.bjorl.2022.04.003

**Published:** 2022-05-20

**Authors:** Renata de Jesus da Silva, Raphaella Ayres Lima Barbosa, Fabio Kenji Okamura, João Gualberto Cerqueira Luz

**Affiliations:** a Hospital M. Dr. Arthur R. de Saboya, Departamento de Cirurgia Oral e Bucomaxilofacial, São Paulo, SP, Brazil; b Fundação Instituto de Pesquisa e Estudo de Diagnóstico por Imagem, São Paulo, SP, Brazil; c Universidade de São Paulo (USP), Faculdade de Odontologia, Departamento de Cirurgia, Prótese e Traumatologia Maxilofaciais, São Paulo, SP, Brazil

**Keywords:** Dentoalveolar abscess, Cellulite, Computed tomography, Length of stay, Hospitalization

## Abstract

**Objectives:**

Odontogenic infections are frequent and can spread, leading to complications such as sepsis and the need for admission to an Intensive Care Unit (ICU). The purpose of this study was to perform a computed tomography analysis of the fascial space involvement and correlate with personal data, laboratory tests, length of hospital stays and admission to the ICU in patients with odontogenic infections who required hospitalization.

**Methods:**

Patients with odontogenic infections admitted between June 2017 and May 2018 were prospectively evaluated. The fascial spaces involved were studied using computed tomography with contrast. The possible correlations of tomographic findings with comorbidities, nutritional status, the causative tooth, laboratory tests, length of hospital stays and admission to the ICU were analyzed.

**Results:**

We identified 66 cases of odontogenic infections which were admitted in the period analyzed. The involvement of primary spaces (86.7%) predominated, followed by secondary ones (8.7%), and cervical (4.6%). The most frequently involved fascial spaces were submandibular (27.1%), buccal (20.8%), sublingual (18.3%), submental (16.7%), and others (17.1%). There were significant differences between laboratory tests (*p* < 0.001), mean hospital stays (*p* < 0.001), and admissions to the ICU (*p* < 0.001) depending on the number of fascial spaces involved. There was no influence of comorbidities, nutritional status, or causative tooth on fascial space involvement.

**Conclusion:**

There was a relationship between greater involvement of fascial spaces assessed by computed tomography and higher values of laboratory tests, more extended hospitalization stays and admission to the ICU.

**Level of evidence:**

Level 2b.

## Highlights

• The involvement of primary spaces predominated in disseminated odontogenic infections.
• Laboratory tests higher values are related to greater involvement of fascial spaces.
• Greater hospitalization stays are related to greater involvement of fascial spaces.
• Intensive care unit admission is related to greater involvement of fascial spaces.

## Introduction

Most cases of odontogenic infections can be treated simply. However, when these infections spread through fascial spaces, they can cause severe morbidities owing to the anatomical proximity of pharyngeal, cervical, thoracic, and intracranial regions, resulting in prolonged hospitalizations, admission to the Intensive Care Unit (ICU), or even death.^1^, ^2^ The most common infections in the head and neck region are those of odontogenic origin.^1^, ^2^

The clinical presentation varies; often, the first symptoms do not reflect the severity of the disease.^3^ Computed Tomography (CT) and laboratory tests such as white blood count and C-Reactive Protein (CRP) are of great value to the professional because they can indicate severity and guide treatment.^4^, ^5^, ^6^

Contrast head and neck CT is indicated in the context of suspected inflammatory infiltration in deep or cervical fascial spaces, involving the lateral pharyngeal, submandibular, sublingual, or masticatory spaces.^4^, ^7^ In addition to highlighting the anatomical spaces involved, CT AIDS pre-surgical planning and localization of the infection focus.^8^, ^9^ CT combines rapid image acquisition and precision of anatomical structures without limitations in the field of view. For these reasons, it is the most reliable technique for assessing profound changes in several fascial spaces.^3^

The purpose of this study was to perform a CT analysis of the fascial space involvement and correlate with personal data, laboratory tests, length of hospital stays and admission to the ICU in patients with odontogenic infections who required hospitalization.

## Methods

We prospectively evaluated patients diagnosed with odontogenic infections who needed hospitalization and were admitted between June 2017 and May 2018 to the Oral and Maxillofacial Surgery Clinic. All patients who participated in the study provided informed consent. The study was approved by the Research Ethics Committee of the Municipal Health Department of São Paulo (SMS/SP) (Protocol No. CAEE: 22418619.2.0000.0086).

Patients were included regardless of age, gender, or race. The presence of signs and symptoms such as dysphagia, odynophagia, mandibular trismus, enlargement of secondary fascial spaces, imminent threat of airway obstruction, need for general anesthesia for treatment, and cases that did not respond well to oral medications were the hospitalization criteria. Exclusion criteria were infections with a non-odontogenic origin, odontogenic infections treated on an outpatient basis, cases not evaluated with tomography at admission, and patients who refused participation.

The age and gender of each patient were recorded, as were comorbidities, as well as harmful habits such as smoking, alcoholism, and drug abuse. Data were also collected regarding patient nutritional status, assessed by the nutrition team of the hospital. They classified patients as eutrophic, mildly malnourished, moderately malnourished, severely malnourished, overweight, and obese.^10^ Next, the clinical manifestations of the disease at hospital admission were noted, including laryngeal crackling and involvement of the mandibular basal, dyspnea, odynophagia, and mandibular trismus. The causative tooth was also considered.

Laboratory tests included complete blood count and C-Reactive Protein (CRP). We also included absolute neutrophil count and the Neutrophil-to-Lymphocyte ratio (N/L). We relied on the hospital laboratory reference values.

Contrast CT scans were used to determine the spread and involvement of the infectious process in the primary, secondary, and cervical fascial spaces. The following spaces were primary: canine, buccal, submandibular, submental, and sublingual; secondary spaces were superficial and deep temporal, masseteric, pterygomandibular, and infratemporal; cervical spaces were lateral pharyngeal, retropharyngeal, and prevertebral. No laboratory and CT exams were performed for research purposes. Data on length of stay and the need for admission to the ICU were assessed.

**Figure 1:**
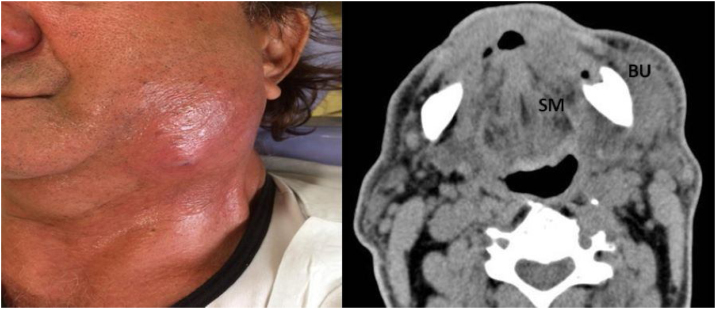
Case # 1. A 74-year-old man without comorbidities presented with an odontogenic infection of the left posterior lower teeth and who was hospitalized for 3 days. (a) Left front view revealing swelling in the lower left third of the face; (b) Contrast enhanced CT revealing a collection in the homolateral Submandibular (SM) and Buccal (BU) spaces.

**Figure 2:**
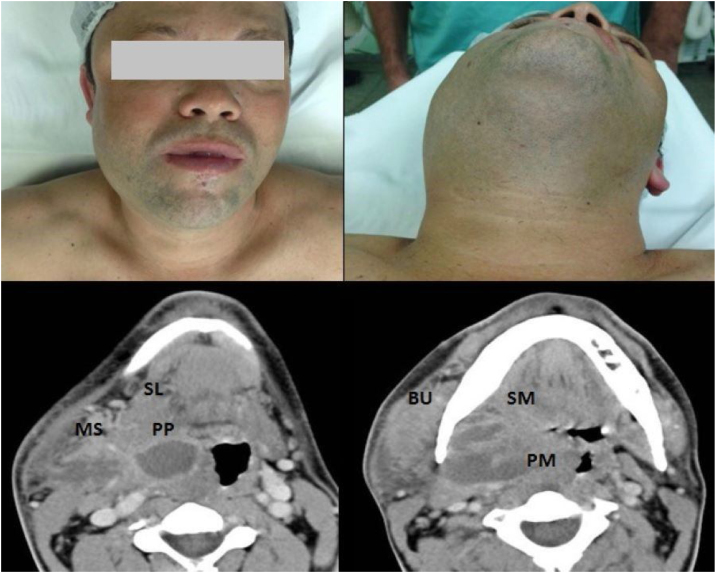
Case # 2. A 49-year-old male smoker presented with an odontogenic infection of the right upper third molar and who was hospitalized for 14 days, including 4 days in the ICU. (a) Areas of swelling in the right hemiface; (b) Areas of swelling in the submandibular region; CT with contrast revealing a collection in the Parapharyngeal (PP) space extending to the Sublingual (SL) and Masseteric (MS) spaces; (d) Collection in the ipsilateral Pterygomandibular (PM), Submandibular (SM) and Buccal (BU) spaces, promoting medial displacement of the right palatal tonsil and luminal reduction of the oropharynx (e) Collection in the right Masseteric Space (MS).

All patients received the same surgical care, including infection drainage from the involved fascial spaces. The most commonly used antibiotics were ceftriaxone combined with clindamycin. According to the severity of the case, the need for artificial ventilation by intubation or tracheostomy was determined with the emergency room staff. Hospital discharge was determined when the clinical parameters or levels of inflammatory markers improved (Figure 1, Figure 2).

The data obtained were subjected to statistical analysis. The likelihood ratio test was applied to assess the relationship between the fascial spaces involved and the variables related to systemic diseases and harmful habits, nutritional status, and causal tooth, as well as between ICU admission and the number of fascial spaces involved. The Kruskal-Wallis test was applied to analyze the possible relationship between the number of fascial spaces involved and the variables related to laboratory tests and hospital stay. Spearman’s correlation analysis was used to determine the degree of relationship between the length of stay and the number of fascial spaces involved. The statistical package IBM SPSS version 25.0 (Statistical Package for Social Sciences, IBM Software Group, Chicago, IL, USA) was used to perform the statistical analyses. Significant differences were defined as *p* < 0.05.

## Results

We identified 78 cases of maxillofacial infections, and 66 cases of odontogenic infections were included in this study. The mean age was 32.7 years. Male sex was predominant with a ratio of 1.4:1. The mean time of hospital stay was 4.3 days. There was an involvement of 240 fascial spaces in the entire cohort, with a mean of 3.63 spaces per patient.

There was a predominance of involvement of primary spaces, with 208 cases (86.7%), followed by secondary ones, with 21 cases (8.7%), and cervical, with 11 cases (4.6%). These were the frequency of involved spaces, in decreasing order: submandibular 65 (27.1%); buccal 50 (20.8%); sublingual 44 (18.3%); submental 40 (16.7%); masseteric 14 (5.8%); canine 9 (3.8%); lateral pharyngeal 9 (3.8%); pterygomandibular 7 (2.9%); and retropharyngeal 2 (0.8%). There were no cases with the involvement of deep temporal, infratemporal, and pre-vertebral fascial spaces.

The occurrence of comorbidities, such as systemic diseases and drug abuse, did not show a pattern regarding the number of fascial spaces involved. Thus, 66.7% of the cases did not have systemic diseases, while 47.0% declared smoking and/or drug abuse. The comparison of the occurrence of systemic diseases and drug abuse regarding the number of fascial spaces involved did not show any significant difference.

Regarding nutritional status, there was a predominance of eutrophy with 40 cases, followed by overweight with 13 cases, mild malnutrition with 9 cases, moderate malnutrition with 2 cases and obesity with 2 cases. The comparison between nutritional status and the number of fascial spaces involved did not show any significant difference. The distribution of nutritional status according to the number of fascial spaces involved is shown in Table 1.

**Table 1:** Distribution of nutritional status and the fascial spaces involved and the significance according to the likelihood ratio test.

Fascial spaces	Nutritional status					Total	*p*-Value
	Moderate malnutrition	Mild malnutrition	Eutrophy	Overwheight	Obesity		
Up to 1	0	0	1	0	0	1	0.597
	0.0%	0.0%	100%	0.0%	0.0%	100%	
2 to 3	1	5	27	8	0	41	
	2.4%	12.2%	65.9%	19.5%	0.0%	100%	
4 to 5	0	2	8	3	2	15	
	0.0%	13.3%	53.3%	20.0%	13.3%	100%	
6 or more	1	2	4	2	0	9	
	11.1%	22.2%	44.4%	22.2%	0.0%	100%	
Total	2	9	40	13	2	66	
	3.0%	13.6%	60.6%	19.7%	3.0%	100%	

Causative teeth were grouped for statistical purposes. Regarding the causal tooth concerning the number of fascial spaces involved, in decreasing order, they were as follows: lower molar in 48 patients, deciduous molar in seven, multiple foci in four, upper premolar in three, upper incisor in three, and upper molar in one patient. The comparisons between the causal tooth and the number of fascial spaces involved did not show any significant differences (*p* = 0.610).

According to the number of fascial spaces involved, the mean values of laboratory tests obtained on hospital admission showed great variation. There was a significant difference in absolute neutrophil counts, N/L ratios, and CRP levels regarding the number of fascial spaces involved; higher values correlated with greater numbers of involved spaces. According to the number of fascial spaces involved, the mean values of laboratory tests obtained at hospital admission are listed in Table 2. Because we found statistically significant differences, the Mann-Whitney test was applied to identify which categories of fascial spaces differed from one another. When comparing these two categories, most comparisons were significant.

**Table 2:** Mean values of laboratory tests obtained on hospital admission according to the number of fascial spaces involved and the significance of the Kruskal-Wallis test.

Laboratory tests	Number of fascial spaces	*n*	Mean	Standard-deviation	*p*-Value
Neutrophils (×10^3^/µL)	Up to 1	1	10.6	0.0	0.001
	2 to 3	41	8.9	3.3	
	4 to 5	15	11.4	6.2	
	6 or more	9	13.7	3.6	
	Total	66	10.2	4.4	
N/L ratio	Up to 1	1	8.2	0.0	<0.001
	2 to 3	41	196.1	923.4	
	4 to 5	15	5.9	3.9	
	6 or more	9	350.2	1025.5	
	Total	66	171.1	815.9	
CRP (mg/L)	Up to 1	1	4.3	0.0	<0.001
	2 to 3	41	7.2	7.0	
	4 to 5	14	12.8	11.4	
	6 or more	9	24.6	11.3	
	Total	65	10.7	10.5	

**Figure 3:**
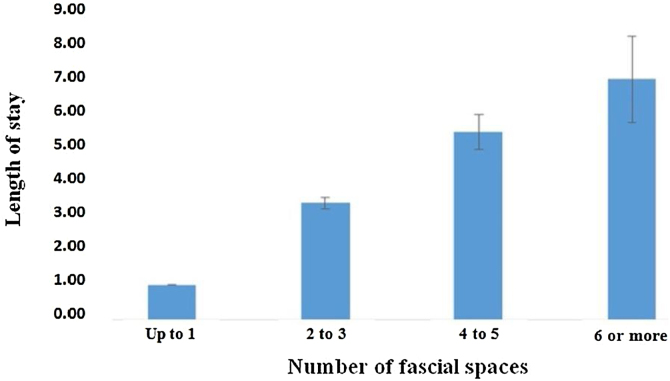
Mean values of the length of hospital stay according to the number of fascial spaces involved.

There was a progressive increase in the mean length of hospital stay according to the number of fascial spaces involved, as follows: up to one space, 1.0 day; two to three spaces, 3.4 days; four to five spaces, 5.5 days; and six or more spaces, 7.0 days. The comparison between the mean length of hospital stays and the number of fascial spaces involved showed a significant difference. The mean values of the hospital stay according to the number of fascial spaces involved are shown in Fig. 3. As statistically significant differences were found, the Mann-Whitney test was applied to identify which categories of fascial spaces differed from one another concerning the length of hospital stay; when comparing these two categories, some comparisons were significant. Next, Spearman correlation analysis was applied to determine the degree of relationship between the length of hospital stay × number of fascial spaces involved. The correlation coefficient (*r*) = +0.613 was found.

Finally, concerning the need for admission to the ICU concerning the number of fascial spaces involved, in decreasing order: six or more spaces in seven patients, four to five spaces in four, and two to three in one patient. The comparison between the need for ICU admission and the number of fascial spaces involved showed a significant difference. The distribution of the need for ICU admission according to the number of fascial spaces involved is shown in Table 3.

**Table 3:** Distribution of the need for admission to the ICU according to the number of fascial spaces involved and the significance of the likelihood ratio test.

Fascial spaces	ICU		Total	*p*-Value
	Yes	No		
Up to 1	0	1	1	<0.001
	0.0%	100%	100%	
2 to 3	1	40	41	
	2.4%	97.6%	100%	
4 to 5	4	11	15	
	26.7%	73.3%	100%	
6 or more	7	2	9	
	77.8%	22.2%	100%	
Total	12	54	66	
	18.2%	81.8%	100%	

ICU, Intensive Care Unit.

## Discussion

We analyzed the involvement of fascial spaces in patients with odontogenic infections who required hospitalization with use of CT and found correlations with laboratory tests, length of hospital stays and admission to the ICU. There were significantly higher absolute neutrophil counts, N/L ratios, and CRP with greater numbers of fascial spaces involved. Also, there was a correlation between the length of hospital stays and the need for ICU admission with the number of affected fascial spaces.

The mean age was 32.7 years. Other authors also found a higher incidence of odontogenic infections in the fourth decade of life.^11^, ^12^, ^13^ Adults 31–50 years old are more likely to neglect oral health.^12^ There was a predominance of males in a proportion of 1.4:1. Other studies also found a higher proportion of males among patients with cervicofacial infections of dental origin.^4^, ^14^, ^15^ The greater prevalence of males might be because they neglect oral health until the condition becomes severe.^12^, ^16^

Imaging exams are crucial for planning the treatment of infections in the cervical region because there is a risk of clinically underestimating their extent and individual characteristics. These tests have five decisive roles: confirming the suspicion of the clinical diagnosis, defining the exact extent of the disease, identifying complications, distinguishing between drainable abscesses and cellulite, and monitoring the progression of the infection to deep neck spaces.^3^ In the present study, CT with contrast allowed the definition of the extent of infection and fascial space involvement, permitting severity assessment.^5^, ^7^, ^17^ Contrast administration improves the ability to differentiate collections and detect vascular complications.^3^ CT is also useful to define the extent and number of anatomical spaces involved, airway obstruction, and visualization of changes in maxillary and mandibular structures.^5^, ^8^, ^18^, ^19^ CT identifies the source of the infection, as osteolytic changes around the causative teeth can be seen, especially when there is trismos.^7^ The routine use of CT in cases of complicated odontogenic infections that present in hospital emergency departments has been questioned because these tests incur higher costs and delay in visits.^20^ Nevertheless, this study demonstrated the importance of CT in evaluating and confirming fascial space involvement, permitting determination of severity and better surgical planning.

The involvement of fascial spaces was most predominant in the submandibular space, followed by buccal, sublingual, submental, masseteric, and other less frequently involved spaces. These frequencies are similar to those reported in other studies.^11^, ^12^, ^21^, ^22^ A study of patients with odontogenic infections who required ICU admission showed a predominance of submandibular, sublingual, and submental spaces.^19^ Deep infection of the cervical space is significantly more frequent in patients with mandibular than those with maxillary odontogenic infections.^4^

More than half of the patients did not report having systemic diseases; however, almost half reported drug abuse. No significant association was found between systemic diseases and drug abuse concerning the number of fascial spaces involved. Studies found that greater numbers of patients who were smokers or drug abusers had more severe odontogenic infections.^5^, ^6^, ^14^ A limitation of the present study involves the possible reticence of patients to report comorbidities, whether due to misinformation or negligence.

Most cases involved lower posterior teeth, especially the third molars, as found in other studies.^5^, ^14^, ^21^, ^23^ Mandibular odontogenic infections with a high risk of involvement of the deep cervical spaces were associated with more extended hospital stays.^4^ However, concerning the causative tooth, no significant association was found with the number of fascial spaces involved.

Concerning nutritional status, more than half of the patients were eutrophic, with no significant difference between the number of fascial spaces and nutritional status. It is noteworthy that overweight patients represented only about 20% of the total. Ours is the first study to analyze possible correlations of nutritional status with fascial spaces in odontogenic infections to the best of our knowledge. Pre-albumin, a sensitive marker for nutritional status, is also a useful marker for determining the severity of odontogenic infections.^1^

Concerning laboratory tests, absolute neutrophil count, N/L ratio, and CRP showed significant differences concerning the number of fascial spaces involved; thus, the greater the number of fascial spaces involved, the greater the severity of the case, which suggests that elevations in these laboratory tests can help determine severity. The inflammatory response provoked during bacterial infections is characterized by neutrophilia and lymphocytopenia. This relationship between inflammatory cells is measured by the N/L ratio, a parameter used to corroborate diagnoses of bacterial infections.^24^ A study showed that ICU admission was significantly associated with higher CRP levels and white blood cell counts.^19^ Positive correlations between the length of stay and leukocytosis, neutrophilia, N/L ratio, and CRP level were shown in patients with odontogenic infections requiring hospitalization.^6^

The mean hospitalization time in the present study was approximately 4 days, similar to the lengths found in other studies.^4^, ^6^, ^25^, ^26^ There was a significant progressive increase in the length of stay according to the number of fascial spaces involved. Some studies found significant relationships between the number of fascial spaces and lengths of hospital stay.^20^, ^22^, ^27^ Other studies suggested the involvement of multiple spaces as a complicating factor in odontogenic infections.^28^, ^29^, ^30^ In patients with odontogenic infections spreading to secondary spaces, there was a significant increase in the length of stay; the likely reason for this increase is worsening of the clinical condition with a higher proportion of life-threatening situations, including the influence of mandibular trismus that complicates removal of the infectious focus.^22^ Also, delay in receiving treatment may explain the greater spread to multiple fascial spaces.^12^

Regarding the need for admission to the ICU, there was an increase in the number of cases according to the greater number of fascial spaces involved. A study conducted with severe odontogenic infections and risk factors for admission to the ICU found the occurrence of edema in the respiratory tract and the need for postoperative mechanical ventilation as one of the factors, with the presence of high levels of CRP associated with the severity of infections in deep spaces as another factor.^19^

## Conclusions

Based on the data collected in this study, it was concluded that there was a relationship between greater involvement of fascial spaces assessed by CT and higher values of laboratory tests, more extended hospitalization stays and admission to the ICU in patients with odontogenic infections who required hospitalization. These findings can help the assistant professional in dealing with these types of infections.

## Ethics

The Research Ethics Committee of the Municipal Health Department of São Paulo (SMS/SP) approved the study (Protocol No. 22418619.2.0000.0086).

## Funding

This research did not receive any specific grant from funding agencies in the public, commercial, or not-for-profit sectors.

## Conflicts of interest

The authors declare no conflicts of interest.
